# Thirst in ICU Patients: A Scoping Review

**DOI:** 10.7759/cureus.84144

**Published:** 2025-05-15

**Authors:** Megumi Horinouchi, Hideaki Sakuramoto, Ayako Fukushima, Shun Yoshihara, Kohei Kajiwara, Megumi Mukoyama, Mayumi Koyanagi, Aiko Mihara, Yuta Imamura

**Affiliations:** 1 Department of Nursing, Hospital of the University of Occupational and Environmental Health, Kitakyushu, JPN; 2 Division of Faculty Development and Nursing, Kindai University, Osaka, JPN; 3 Department of Nursing, Hokkaido University of Science, Sapporo, JPN; 4 Department of Critical Care and Disaster Nursing, Japanese Red Cross Kyushu International College of Nursing, Munakata, JPN; 5 Faculty of Nursing, Shimonoseki City University, Shimonoseki, JPN; 6 Department of Nursing, Japanese Red Cross Fukuoka Hospital, Fukuoka, JPN; 7 Department of Nursing, Fukuoka Tokushukai Hospital, Kasuga, JPN; 8 Department of Nursing, Kurume University Hospital, Kurume, JPN; 9 Department of Nursing, National Hospital Organization Kumamoto Medical Center, Kumamoto, JPN

**Keywords:** critical care, intensive care unit, scoping review, symptom, thirst

## Abstract

Thirst is one of the most frequently experienced symptoms among patients in intensive care units. Previous reviews of thirst in ICU patients and interventions to alleviate thirst had certain limitations. Therefore, we aimed to systematically explore and map the literature on the prevalence, risk factors, specific measurement methods, and intervention strategies for thirst in ICU patients and to identify areas where further research is needed. A scoping review was conducted to limit the search to studies published by August 2, 2024. A literature search was performed using PubMed, CINAHL, CENTRAL, and Igaku Chuo Zasshi databases. Two reviewers independently screened the literature according to the inclusion and exclusion criteria and extracted data from the selected studies. A total of 53 studies met the eligibility criteria. The combined prevalence of thirst, reported in 11 studies, was 66% (95% CI: 56-76). Four studies reported the risk factors for thirst, including sepsis, gastrointestinal disease, analgesics, diuretics, hyperglycemia, and elevated serum sodium levels, through multivariable analysis. The intensity of thirst and degree of distress were measured using the Numerical Rating Scale, the Visual Analog Scale, and the Likert scale in many studies. The interventions primarily consisted of oral and lip moisturization via sprays and swabs with cold water, many of which were bundled or packaged. The frequency of moisturization interventions for thirst varied from every 30 minutes over a three-hour period to three times a day, and it remained unclear which intervention frequency was the most effective. Other interventions included early oral intake, humidification, and use of music and virtual reality. None of the studies included interventions, such as medication adjustments or electrolyte correction, despite these being recognized as potential risk factors. Future studies should validate the measurement methods and develop objective measurement tools. The intervention types and frequencies that are most effective for treating thirst in ICU patients are unclear. Therefore, further research is required to evaluate the type, timing, and frequency of interventions while considering the identified risk factors.

## Introduction and background

Thirst is described as the “longing for fluids” and is a conscious and subjective experience [1]. It plays a critical role in maintaining the body’s fluid and electrolyte balance. This sensation is not simply a response to dryness in the mouth or throat, but the result of a complex physiological process. Specifically, thirst is regulated by an intricate system of neurohormonal and ionic signaling that monitors changes in the body’s water and sodium levels. When these levels become unbalanced - for example, due to dehydration or elevated sodium concentration - the brain activates the sensation of thirst to prompt fluid intake and restore homeostasis [2]. Those receiving mechanical ventilation are particularly affected, as endotracheal tubes prevent lip closure, contributing to oral dryness and making thirst a prominent, distressing symptom [3]. The magnitude of this issue is supported by data showing that over 70% of critically ill adult ICU patients with high illness severity (APACHE II score ≥20) experience thirst [4]. This finding was observed in patients who had been admitted to the ICU for at least three days with severe conditions such as cardiac/respiratory failure, liver cirrhosis, sepsis with multiple organ dysfunction, or malignancy-associated organ failure. Thirst is also reported by ICU patients to be one of the most frequent and intense symptoms, alongside pain [5], and is recalled by ICU survivors as the most distressing experience during their ICU stay [6].

In 2024, a scoping review of thirst in ICU patients was published [7]. This review summarizes the potential causes, risk factors, diagnostic and measurement tools, potential co-occurrence with other distressing symptoms, and management of thirst in ICU patients. However, the scoping review only included studies that directly measured thirst in ICU patients, excluding those that broadly evaluated stress or discomfort. Therefore, studies that evaluated thirst as a secondary outcome or unexpected finding were not included in the scoping review. Additionally, the scoping review limited its search to publications in English, Norwegian, Swedish, Danish, and Dutch. However, there are numerous reports on thirst published in languages such as Chinese, which indicates that the search may not have been comprehensive. In addition, the scoping review included both thirst as a subjective symptom and objective dry mouth, such as the wetness tester [7]. However, previous research has demonstrated distinct differences between ratings of thirst and dry mouth before and after oral care in intensive care patients [8]. Therefore, our review specifically focused on the concept of thirst, deliberately excluding literature solely addressing dry mouth.

Furthermore, a systematic review of interventions to alleviate thirst reported various strategies, including cooling sprays, cotton swabs, lip moisturizers with menthol, and the use of humidifiers [9]. However, the search did not include various interventions, such as the use of virtual reality. Furthermore, the systematic review covered only intervention studies conducted up to 2020. Although randomized controlled trials addressing thirst have been conducted [10,11], no subsequent systematic review has incorporated this newer evidence. Due to the considerable heterogeneity and lack of consensus in the literature on thirst among ICU patients, we chose to conduct a scoping review. This approach allows for a comprehensive overview of the current body of knowledge, clarifying key themes and evidence gaps that require further investigation.

Therefore, an updated and comprehensive scoping review that includes a broader spectrum of interventions and critically ill patient populations is warranted to identify current gaps between research evidence and clinical practice. In this scoping review, we systematically explored and synthesized the literature on the prevalence of thirst, associated risk factors, assessment tools, intervention strategies, and their reported effects in ICU patients, while also highlighting areas where further research is needed.

## Review

Methods

Design and Protocol Registration

This scoping review was conducted following the methods outlined by Arksey and O'Malley [12], the revised recommendations by Levac et al. [13] and Colquhoun et al. [14], and the Preferred Reporting Items for Systematic reviews and Meta-Analyses (PRISMA) Scoping Review Extension guidelines [15]. The protocol for this study was registered on the Open Science Framework and can be accessed at https://osf.io/czw3n (last registered date: October 23, 2024). The analysis method was considered based on the search results, and the protocol was registered in October during the search.

Research Questions

The research questions were as follows: “What are the characteristics of patients who experience thirst (e.g., demographic, treatment, and physiological factors)?”, “What are the methods to measure thirst?”, “What is the prevalence of thirst during ICU admission?”, “Is there any relationship between the symptom rate and the clinical course of the patients after admission to the ICU?”, “What are the factors of thirst exacerbation and relief?”, and “What interventions are effective for relieving thirst?”

Search Strategy

PubMed, CINAHL, CENTRAL, and Igaku Chuo Zasshi (Ichushi-Web) databases were searched for studies published from their inception to August 2, 2024. The keywords used were thirst, intensive care, and their synonyms. The search strategy is presented in Appendix A.

Eligibility Criteria

The concept of interest was thirst, and literature on dry mouth alone was not included. Because some reports have indicated that dry mouth does not correlate with thirst [8], the present study focused on thirst as a subjective symptom in patients. The inclusion and exclusion criteria are shown in Table 1.

**Table 1:** Inclusion and exclusion criteria for the scoping review

Inclusion	Exclusion
(1) Patients: Critically ill adults	(1) Studies describing only dry mouth, without reporting information on thirst
(2) Concept: Thirst (including causes, risk factors, diagnosis, measurement, symptom intensity, prevalence, interaction, and management)	(2) Publication type: Reviews, case reports, opinion pieces, qualitative studies, books, letters, oral presentations, posters, and studies with only an abstract available
(3) Context: ICU	
(4) Study type: Any article discussing thirst and its risk factors, assessment methods, or interventions to alleviate thirst in ICU patients	
(5) No language limitations	
(6) No date of publication limitations	

Study Selection and Data Extraction

Citations were uploaded to Rayyan (http://rayyan.qcri.org), and duplicates were removed. The titles and abstracts were independently reviewed by two reviewers during primary screening. For secondary screening, the full text was uploaded to Rayyan, and two reviewers independently evaluated the full text based on the inclusion and exclusion criteria. Any conflicts were discussed and resolved until a consensus was reached. If necessary, a third reviewer was involved for arbitration. Data extraction was standardized, and the study characteristics (author, year of publication, country, language, ICU setting, participants, ventilator management status, study design, study objectives, intervention method, intervention timing, and intervention duration) and main outcomes (symptom rate, thirst intensity, thirst distress, intervention effect, and risk factors) were extracted from each study. Since scoping reviews typically do not include a formal risk of bias assessment [15], we did not perform one in this study.

Data Synthesis

The search results are presented using a PRISMA flow chart. Quantitative data were integrated using descriptive and inferential statistics and are presented in tabular form. The data are summarized in graphical and tabular formats (numerical summaries) and narrative formats (descriptive summaries). Multiple studies were analyzed and synthesized according to the research questions, and the results were summarized. Descriptive statistics for symptom rates and intensity are reported as means (standard errors) and medians (interquartile ranges). Symptom rates were analyzed using the Stata software (version 18; Stata Corp, College Station, TX, USA). The pooled prevalence and 95% CIs were estimated based on the number of events in each group. Heterogeneity between studies was quantified using visual inspection of forest plots, Cochran’s Q statistic (p < 0.05), and the I² statistic. The I² statistic estimates the percentage of observed variability between studies owing to heterogeneity rather than chance, ranging from 0% to 100% (with values of 25%, 50%, and 75% indicating low, moderate, and high heterogeneity, respectively). In this review, I² values exceeding 75% were considered indicative of significant heterogeneity, and a random-effects model was used to adjust for observed variability [16,17].

Results

Study Selection

**Figure 1:**
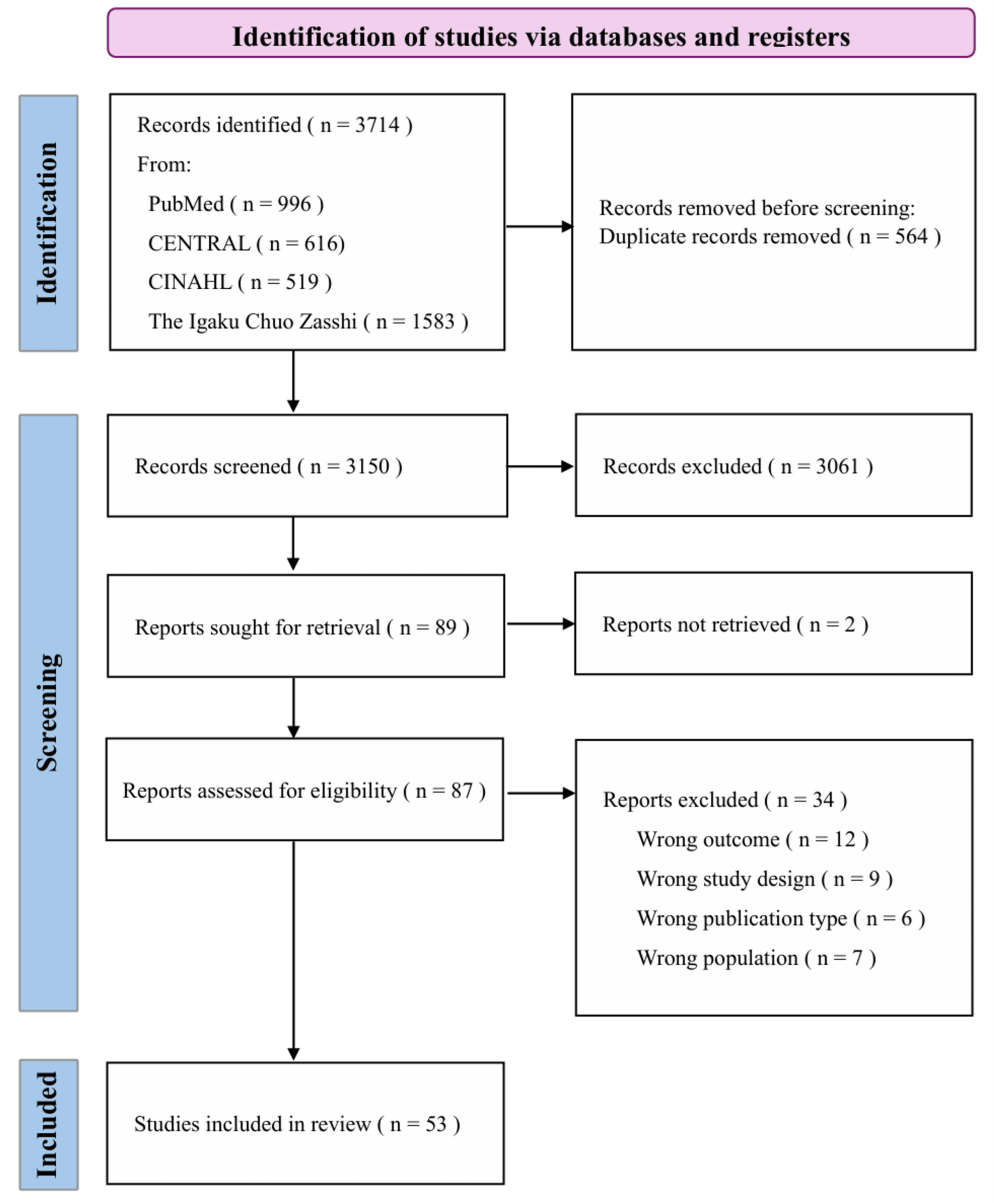
PRISMA flow diagram of the study selection CINAHL, Cumulative Index to Nursing and Allied Health Literature; PRISMA, Preferred Reporting Items for Systematic reviews and Meta-Analyses

The search identified 3,150 articles. After screening the titles and abstracts, 89 articles were shortlisted, of which two were not accessible. Thus, 87 full-text publications were assessed for eligibility through a secondary screening. Finally, 53 studies were included in this review. A flowchart of the study selection process is shown in Figure 1.

Study Characteristics

The characteristics of the included studies are presented in Table 2 and Table 3. Twenty studies were conducted in Asia [8,10,11,18-34], 11 in North America [4,6,35-43], one in South America [44], and 21 in Europe [5,45-64]. Thirty-seven of the 53 studies included patients who received mechanical ventilation [4-6,8,19,21-26,29,33,36-39,41-54,56,57,59,60,62,63]. A total of 29 non-intervention studies were included (Table 2) [4-6,8,18-23,35-39,44-57]. A total of 24 studies focused primarily on thirst [8,10,11,18,22,24-31,33,34,37,40,42,43,45,58,59,61,64], 25 studies addressed discomfort and stress in the ICU [4-6,19,20,23,32,35,36,38,39,44,46-53,56,57,59,62,63], and four studies focused on other topics such as delirium [21,54,55,60]. A total of 24 intervention studies were included (Table 3) [10,11,24-34,40-43,58-64]. The types and methods of interventions showed significant heterogeneity. Among the intervention studies, 15 were randomized controlled trials [10,11,24-29,40,41,58-62], and seven were non-randomized trials [30,31,32,34,43,63,64]. One study was a quality improvement project report [42], and the other was a bench study [33].

**Table 2:** Characteristics of noninterventional studies ARICU, anesthesiology and reanimation ICU; CAM-ICU, Confusion Assessment Method for the Intensive Care Unit; ECMO, extracorporeal membrane oxygenation; EICU, electronic ICU; GEE, generalized estimating equation; HFNC, high-flow nasal cannula; ICUESS, ICU Environmental Stressor Scale; ICU-SEQ, ICU stressful experience questionnaire; IMV/NIV, invasive mechanical ventilation/noninvasive mechanical ventilation; IPREA, Inconforts des Patients de REAnimation; MICU, medical ICU; MV, mechanical ventilation; NRS, Numeric Rating Scale; RASS, Richmond Agitation Sedation Scale; SICU, surgical ICU; TDS-HF, Thirst Distress Scale for patients with heart failure; VAS, Visual Analog Scale; VV-ECMO/VA-ECMO, veno-venous ECMO/veno-arterial ECMO

First author, year, and country	Study design	Setting	Sample (N)	Inclusion criteria	Research objective	Measurement method and timing	Key results
Saltnes-Lillegård, 2023, Norway [5]	Observational multicenter study	Six SICUs and MICUs at two facilities	353 (first day: 195)	Age ≥18, patients who need MV, need for continuous vasoactive therapy, or ICU stay >24 hours	Describe the prevalence, intensity, and distress of five symptoms in ICU patients and investigate possible predictive factors associated with symptom intensity	Symptom intensity: 11-point Likert Scale (NRS), symptom distress: 11-point Likert Scale (NRS), measured daily during ICU stay (seven days)	First day at ICU: 66% reported thirst as the most common symptom and the highest mean intensity score (6.13). Thirst was the most common (64%) and strongest symptom (mean score of 6.05) during the seven days in the ICU. Multivariate GEE analysis showed that analgesic administration (B = 0.88, 95% CI: 0.18-1.59) and sepsis diagnosis (B = 1.71, 95% CI: 0.58-2.84) were associated with increased thirst intensity over a seven-day period.
Negro, 2022, Italy [45]	Observational study	Mixed ICU, cardiac ICU, and neurosurgery ICU	220	Age ≥18, GCS ≥9, spontaneous breathing with tracheal intubation or tracheostomy	Determine the incidence and intensity of thirst in patients admitted to the ICU and its association with airway devices, airway humidification, patients’ characteristics, and therapy	Thirst Intensity: NRS, measured daily during ICU stay	Thirst was seen in 76.1% of observations, with an average of 5.37 in strength. NRS ≥8 was 26.5%, NRS ≥5 was 52.2%, and NRS = 0 was 24.1%. Thirst intensity was predicted by high doses of diuretics (> 100 mg/day), increasing serum sodium concentration, absence of oral hydration, and the presence of xerostomia.
Doi, 2021, Japan [8]	Observational study	Mixed ICU	86	Age ≥20, no disorientation/delirium	Investigate the impact of oral care on thirst perception and dry mouth assessments	Thirst intensity: NRS, measured before and after oral care and every hour until four hours after oral care	Thirst decreased post-oral care but lasted one hour; NRS (thirst intensity) did not correlate with oral moisture or the modified revised oral assessment guide
Gültekin, 2018, Turkey [46]	Observational study	SICU and ARICU	98	Age ≥18, ICU stay ≥24 hours	Describe environmental and psychological stressors affecting ICU patients and determine their priorities	Turkish ICU Environmental Stressor Scale Assessment, Stress: 4-point Likert scale	Thirst was the largest stressor among all patients (average: 2.44). Thirst (mean: 2.61) was also the biggest stressor in postoperative patients. Patients with internal diseases cited thirst as the biggest stressor.
Saltnes-Lillegård, 2024, Norway [47]	Prospective cohort study	Six mixed ICUs	353	Age ≥18, need for IMV/NIV, need for continuous angio-agonists, or need for ICU stay ≥24 hours	Identify and compare subgroups of ICU patients	Five of the 10 symptoms of ESAS, including thirst, were evaluated, measured once daily (up to seven days)	Three symptom classes were identified, and thirst was a common symptom in all classes. Middle-class patients (n = 177, 50.1%) had a high prevalence of thirst and fatigue.
Sato, 2023, Japan [18]	Prospective cohort study	Mixed ICU	100	Age ≥20, GCS ≥14, post-extubation ICU stay ≥24 hours	Assess the effect of post-extubation HFNC on thirst	Thirst Intensity: NRS, measured four and 24 hours after extubation	After adjustment, HFNC was significantly associated with a decrease in thirst intensity at 24 hours after extubation (adjusted OR: 0.14, 95% Cl: 0.04-0.49, p = 0.002) and a decrease in thirst intensity at four hours after extubation (adjusted OR: 0.19, 95% Cl: 0.06-0.60, p = 0.005).
Kalfon, 2010, France [48]	Prospective cohort study	MICU and SICU	868	Adult ICU survivors	Develop and validate the IPREA questionnaire for the assessment of discomfort related to ICU stay	Evaluated for 16 symptoms of IPREA, discomfort: VAS, measured on the day of ICU discharge	Thirst was the fourth highest-scoring discomfort (mean ± SD: 32 ± 34); 17.9% of patients complained of thirst discomfort with a VAS score of ≥ 70.
Gunnels, 2024, United States [35]	Descriptive cross-sectional study	SICU, EICU, cardiac ICU, and transplant ICU	180	Age ≥18, RASS 0, CAM-ICU negative, ICU stay ≥24 hours	Assess patient-reported discomfort among critically ill patients	18-item IPREA rating, symptom discomfort: 11-point Likert Scale (NRS), single measurement at study enrollment	Of the 18 items, thirst discomfort scores were the third highest (mean: 3.3), which was equivalent to pain.
Peterson, 2023, United States [36]	Descriptive cross-sectional study	SICU, MICU, transplant ICU, and multispecialty ICU	114	Age ≥18, RASS -1 to +1, CAM-ICU negative, use of NIV in the previous 24 hours	Identify the presence, intensity, and distress of symptoms in patients receiving NIV in the ICU using MESAS	MESAS: Yes/no, symptom intensity: 3-point Likert scale, symptomatic distress: 3 -3-point Likert scale, single measurement at study enrollment	Thirst was the most frequently reported (75.4%). Among participants who reported thirst, 47% rated its intensity as severe, and 45% rated its distress as severe.
Karaer, 2021, Turkey [49]	Descriptive cross-sectional study	SICU	120	Age ≥18, ICU stay: 24-72 hours	Determine environmental stressors perceived and their level of satisfaction with nursing care	4-point Likert Scale with ICUESS, measured after ICU discharge	Thirst was the second highest stressor (mean: 2.79).
Zengin, 2019, Turkey [50]	Descriptive cross-sectional study	General ICU	116	Age ≥18, ICU stay ≥24 hours, GCS assessment orientation: normal	Examine the relationship between stressors and patients’ experiences in the ICU	Intensive Care Experience Scale, stress distress: 5-point Likert scale, measured before ICU discharge	Of the respondents who reported experiencing extreme stress, 50.9% said that it was caused by thirst, which was the most common cause. There was a moderate positive relation between the stressors, noise (r = 0.534; P).
Takashima, 2017, Japan [19]	Descriptive cross-sectional study	SICU	96	Age ≥20, CAM-ICU negative, MV management ≥ 12 hours	Clarify the actual state of stress experience and related factors in ICU patients who have been mechanically ventilated for >12 hours	ICU-SEQ, stress level: 6-point Likert scale, measured before ICU discharge	The combined percentage of patients with moderate to extreme thirst was 76%. The only factor associated with thirst was CRP level at the time of discharge from the ICU (r = 0.23, p = 0.026).
Stotts, 2015, United States [37]	Descriptive cross-sectional study	MICU, cardiac ICU, and neurosurgery ICU	353	Age ≥18, ICU stay ≥ 24 hours, RASS -1 to +1, CAM-ICU negative	Identify predictors of the presence, intensity, and distress of thirst in ICU patients	Thirst intensity: NRS, thirst pain: NRS, measured once at study enrollment	Thirst was predicted by high doses of opioids, high doses of furosemide, selective serotonin reuptake inhibitors, and low ionized calcium. Predictors of thirst intensity: lack of oral fluid intake and diagnosis of digestive disorders; predictors of thirst distress: MV, negative fluid balance, antihypertensive drugs, and gastrointestinal or “other” diagnosis.
Rose, 2014, Canada [6]	Descriptive cross-sectional study	General ICU	27	MV use for >21 days in ICU	Compare ICU and weaning center memories, and examine the link between delusional memories and psychological outcomes	ICU Memory Tool, ICU-SEQ evaluation, measured after discharge from the hospital	Of the 23 people who had memories of both the ICU and the specialized withdrawal center, 16 (70%) remembered feeling thirst in the ICU. Of those 16 (88%), 14 had memories of thirst discomfort.
Hweidi, 2007, Jordan [20]	Descriptive cross-sectional study	Three cardiac ICUs	165	ICU stay ≥24 hours	Identify the principal physical and psychological stressors as perceived in ICUs	ICUESS Assessment, Distress: 4-point Likert scale, measured on the second to third day after ICU discharge	Thirst was the fourth largest factor in stress (mean ± SD: 3.31 ± 0.79).
Ayllón Garrido, 2007, Spain [51]	Descriptive cross-sectional study	General ICU	91	Age ≥18, ICU stay ≥ 3 days	Describe the stressful environmental events	Ballard’s Environmental Stressors Scale, Stress: 4-point Likert scale, measured three days after ICU discharge	Thirst was the most stressful factor, felt by 62.6%.
Cazorla, 2007, France [52]	Descriptive cross-sectional study	General ICU	70	Ventilator management ≥24 hours	Analyze patients’ assessment of the quality of care in the ICU	A nine-item, 20-question questionnaire, questionnaire was sent post-discharge	54% remembered mechanical ventilation; thirst was the second most common factor that plagued patients in the ICU environment (39%).
Jelen, 1979, Germany [53]	Descriptive cross-sectional study	SICU and EICU	30	ICU stay ≥5 days	Investigate psychological experiences in the ICU	Interview based on questionnaire, measured six to 24 months after ICU discharge	More than 25% of patients experienced strong thirst during ICU stay.
Krupa, 2021, Poland [54]	Cross-sectional pilot study	Cardiac ICU	32	Age ≥18, equipped with VV-ECMO/VA-ECMO	Show the incidence of delirium in patients after ECMO therapy and factors affecting the occurrence of delirium	Thirst intensity: Thirst Intensity Scale (0 points: no thirst, 10 points: unbearable thirst), measured before and after ingestion of ice	There was no association between delirium and thirst during ECMO. The intensity of thirst before administration of the ice piece was 8.34 ± 1.36 (median: 8) and was significantly reduced (p < 0.008) to 7.16 ± 2.05 after the administration of the ice piece.
Sato, 2019, Japan [21]	Retrospective cross-sectional study	Mixed ICU	401	Age ≥18, RASS -1 to +1	Investigate the association between thirst and delirium	Thirst intensity: NRS (NRS ≥8 is defined as strong thirst), measured at least twice a day	40.6% of patients experienced strong thirst during their stay in the ICU. Logistic regression analysis showed that patients with persistent strong thirst had a higher risk of delirium than patients without persistent strong thirst (OR = 4.95, 95% CI: 2.58-9.48, p < 0.001).
Lin, 2023, China [22]	Prospective descriptive study	SICU, EICU, MICU, and cardiac ICU	301	Age ≥18, RASS -1 to +1, ICU stay ≥24 hours	Investigate the symptomatic rate of thirst and associated risk factors	Thirst Intensity: NRS, 3-point Likert scale in patients with NRS ≥ 3, one measurement at 6 pm after 24 hours of ICU stay	Thirst was reported by 69.8% patients; Risk factors for thirst: nil per os order (OR = 4.10, 95% CI: 1.44-11.69), surgery (OR = 2.96, 95% CI: 1.11-7.93), high glucose (OR = 3.36, 95% CI: 1.01-11.17), greater disease severity (OR = 1.13, 95% CI: 1.02-1.24).
Puntillo, 2010, United States [4]	Prospective descriptive study	Two general ICUs	171	Age ≥18, ICU stay ≥3 days	Provide a focused, detailed assessment of the symptom experiences of ICU patients at high risk of dying, and evaluate the relationship between delirium and patients' symptom reports	ESAS (10 symptoms): Yes/no, symptom intensity: 3-point Likert scale, Symptomatic distress: 3-point Likert scale, measured up to 14 days, up to seven times every other day	Thirst was the second most common symptom (70.8%) and the strongest symptom (mean intensity: 2.16, SE: 0.087).
Dessotte, 2016, Brazil [44]	Prospective correlational study	General ICU	105	Age ≥18, postoperative patients who underwent coronary artery bypass grafting or mitral valve surgery	Investigate stressors perceived by patients in the immediate postoperative period of cardiac surgery and their association with sociodemographic and clinical characteristics	Portuguese Environmental Stressor Questionnaire Assessment, Stress: 5-point Likert scale, measured within 48 hours after discharge from the ICU	The item “being thirsty” was evaluated as the most stressful (mean ± SD: 2.6 ± 1.0). Thirst was not stressed: 19.0%, moderately stressed: 24.8%, very stressed: 32.4%, extreme stress: 23.8%.
Siami, 2013, France [55]	Prospective interventional study	ICU	30	Septic shock patients	Investigate whether AVP response changes in patients recovering from septic shock	Thirst intensity: VAS, measured before and after osmotic loading	Physiological saline load was performed. Non-responders were defined as those with a slope of the relation between AVP and plasma sodium levels <0.5 ng/mEq, and thirst perception was significantly diminished in non-responders.
Flim, 2022, Netherlands [56]	Validation study	Mixed ICU	56	Age ≥18, RASS -1 to +1, CAM-ICU negative, ICU stay ≥ 24 hours	Determine the validity and reliability of the “TDS-HF” for thirst distress in ICU patients	Thirst intensity: NRS, thirst distress: TDS-HF/NRS, single measurement	Content validity was low, with an item content validity index between 0.25 and 0.75. The simultaneous validity was high, with Spearman’s correlation coefficient between TDS-HF and NRS for thirst distress of 0.71.
Wang, 2015, China [23]	Retrospective study	Cardiac ICU	800	Age ≥18, MV time ≥4 hours	Analyze major complaints from patients during mechanical ventilation after cardiac surgery	Degree of discomfort: VAS, measured within 24 hours of ICU admission	Thirst and dry mouth were among the independent factors that caused discomfort in patients during MV management after cardiac surgery.
Baumstarck, 2019, France [57]	Secondary analysis	Mixed ICU, SICU, and MICU	994	Age ≥18 years, ICU stay ≥3 days	Validate the 18-item version of the IPREA questionnaire	18-item IPREA rating, Discomfort: VAS, measured on the day of ICU departure	Thirst was one of the three items with the strongest discomfort among the 18 items (mean ± SD: 31 ± 35.04).
Li, 2006, United States [38]	Pilot study	SICU and EICU	15	Age 21-80, orientation: normal, MV ≥ 12 hours	Document the prevalence and intensity of nine symptoms and examine the relationships among these symptoms	Measures of nine symptoms, symptom intensity: NRS, measured daily	Thirst was the strongest of the nine symptoms (mean ± SD: 5.7 ± 3.7); 80% felt thirst, and 40% felt severe (NRS ≥ 7) thirst.
Nelson, 2001, United States [39]	Prospective study	MICU	100	Adult critically ill cancer patients	Characterize the symptom experience of a cohort of ICU patients at high risk for hospital death	ESAS assessment, thirst intensity: 4-point Likert scale	71% of ESAS respondents reported experiencing moderate-severe unsatisfied thirst.

**Table 3:** Characteristics and effectiveness of intervention studies on thirst ↓ = improvement; ↑ = worsening; → = no significant change ANP, Anesthesiological Questionnaire for patients after anesthesia; COHG, conventional oral hydration; EOH, early oral hydration; HFNO, high-flow nasal oxygen; JCS, Japan Coma Scale; NA, not applicable; RCT, randomized controlled trial; SEDAICU, Stress Factors in Intensive Care Unit Questionnaire; SOFA, Sequential Organ Failure Assessment

First author, year, and country	Study design	Setting	Sample (N)	Inclusion criteria	Research objective	Measurement method	Intervention methods	Intervention frequency and timing	Duration of the intervention	Effect of thirst intensity	Effect of thirst distress
Gungor, 2024, Turkey [58]	RCT	SICU	110 (Experimental group: 55/Control group: 55)	Age 18-65, post-abdominal surgery, fasting ≥6 hours, Thirst Intensity NRS ≥ 3	Investigate the effects of intraoral cold water spray in patients having abdominal surgery	Thirst intensity: NRS; measured at 1, 4, 8, and 16 hours after surgery	Spraying cold water (4°C) (three times per hour)	NRS ≥3 at 1, 4, 8, and 16 hours postoperatively	Postoperative 16 hours	↓	NA
Lian, 2024, China [10]	RCT	SICU	56 (Experimental group: 28/Control group: 28)	Age 18-80, ICU stay ≥6 hours, fasting after extubation	Assess the effect of ice-cold water spray applied for postoperative thirst and to establish a framework for mitigating thirst in ICU patients	Thirst intensity: NRS, Oral comfort: NRS, measured at 0.5 hours before and after each intervention	After spraying with ice water (0-6°C), moisturize the lips with a cotton swab dipped in paraffin oil	Four times during six hours after extubation	Six hours	↓	↓
Wu, 2024, Taiwan [24]	RCT	MICU	36 (cold saline spray group: 18/cold distilled water spray group: 18)	Age ≥20, RASS -1 to +1, during tracheal intubation, MV ≥ 24 hours, thirst intensity NRS ≥ 3	Compare the effectiveness of cold saline spray and cold distilled water spray in relieving thirst in patients with endotracheal tubes placed in the ICU	Thirst intensity: NRS, measured before and after three interventions	Each intervention is 15 minutes. Cold saline group: spray cold saline (2-8°C), Cold distilled water group: spray cold distilled water (2-8°C)	Three times between 20:00 and 22:30, with an interval of 30 minutes	Three days	↓	NA
Luo, 2023, China [25]	RCT	Mixed ICU	113 (Group A: 38/Group B: 39/Group C: 36)	Age 18-80, RASS -1 to +1, oral tracheal intubation time ≥24 hours, Thirst intensity NRS ≥3	Investigate the effect of different doses of ice water spray on thirst degree and complications in ICU patients with MV	Thirst intensity: NRS, thirst distress: Thirst Distress Scale (0: no pain to 10: very painful), measured every four hours during the intervention time (post-intervention)	Group A: infusion of 1.5 mL of room temperature drinking water into the oral cavity, Group B: spray 1.5 mL ice water (0-4°C) into the oral cavity, Group C: spray 0.5 mL ice water (0-4°C) into the oral cavity	Every two hours, between 7:00 and 23:00	Until extubation (two to three days)	↓	↓
Ding, 2023, China [26]	RCT	SICU	80 (Intervention group: 40/Control group: 40)	Post-surgery, ICU stay ≥48 hours, oral tracheal intubation, thirst intensity NRS ≥3	Determine the clinical efficacy of an intervention using a combination of wiping and spraying with peppermint alcohol solution for ICU patients	Thirst intensity: NRS, thirst distress: Thirst Distress Scale, measured before and 24 hours after intervention	Oral care with cold peppermint alcohol solution, oral spraying of cold menthol solution, application of menthol lip balm on the lips	Oral care: every six hours, start spraying and lip moisturizing after extubation (seven times/day)	48 hours	↓	↓
Lin, 2022, China [27]	RCT	Cardiac ICU	145 (Group A: 47/Group B: 47/Control group: 49)	Patients who underwent cardiac surgery and received MV	Evaluate the safety, feasibility, and effects of a spray-based oropharyngeal moisturizing program for cardiac surgery patients following endotracheal extubation	Thirst intensity: NRS, thirst discomfort: numerical rating from 0 to 14; measured before intervention, three hours after extubation, and six hours after extubation	Group A: low-temperature cooling spraying, Group B: low to normal temperature spraying (both Group A and Group B sprayed twice an hour), control group: if the patient complained of thirst, oral moisturizer was applied with a moistened cotton swab.	Intervention starts between zero and six hours after extubation. Intervention two times/hour, then at the patient’s request	During the first six hours of intervention	↓	↓
Liang, 2022, China [28]	RCT	Cardiac ICU	84 (EOH group: 39/COH group: 41)	Age ≥18, postoperative cardiac surgery	Investigate the effect of oral rehydration one hour after extubation in patients undergoing cardiac surgery	Thirst intensity: NRS, measured before intervention and every hour after extubation (four hours)	EOH group: 30 mL of warm water one hour after extubation, followed by drinking 50 mL of warm water per hour for four hours, COH group: no oral intake for four hours after extubation	One to four hours after extubation	Four hours after extubation	↓	NA
Zhang, 2022, China [11]	RCT	Mixed ICU	61 (Experimental group: 31 people/Control group: 30 people)	Age ≥18, ICU stay ≥24 hours, during fasting management, thirst intensity NRS ≥ 3	Demonstrate the effectiveness of an intervention bundle to relieve thirst and dry mouth	Thirst intensity: NRS, measured at 8:00 am before intervention and at 6:00 pm after intervention (three days)	Experimental group: intervention bundle, vitamin C spray, mouthwash with 40°C peppermint water, lip moisturizer (main ingredient: glycerin); control group: placebo group, saline spray, mouthwash with 40°C mouthwash, lip moisturizer with water	Spray: every hour, mouthwash: once at 2 pm, lip moisturizer: every two hours	Three days (8 am to 6 pm)	↓	NA
Merliot-Gailhoustet, 2022, France [59]	Crossover RCT	SICU	60 (50 completed all relaxations)	Age ≥18, RASS ≥0, CAM-ICU negative, SOFA score ≥3	Assess the impact of different electronic relaxation devices on common stressful patient symptoms experienced in the ICU	Thirst intensity: NRS, measured before and after each intervention	Four relaxation sessions in a crossover design: standard relaxation (TV/radio), music therapy, real video images (VR system), and synthetic video images (VR system)	At least one hour washout time between each session	15 minutes for each session	→	NA
Zhang, 2021, China [29]	RCT	Mixed ICU	60 (Observation group: 30/Control group: 30)	Age 18-80, RASS -1 to +1, Oral tracheal intubation ≥24 hours, thirst intensity NRS ≥3	Investigate the effect of ice water spray on thirst in patients undergoing transoral intubation	Thirst intensity: NRS, measured daily after seven interventions	Observation group: spray of ice-sterilized water (0-6°C), Control group: lip moisturizer with a swab soaked in water	Seven times/day and at the patient’s request	Until extubation	↓	NA
Ford, 2020, United States [40]	RCT	Cardiac ICU	149 (Early oral hydration group: 75/Usual group: 74)	Postoperative cardiothoracic surgery	Determine the effect of early oral hydration on adverse events and thirst in patients after cardiothoracic surgery	Thirst intensity: NRS; measured immediately after extubation, six hours after extubation and 12 hours after extubation	Early oral fluid intake group: patients who meet criteria ingest ice chips two hours after extubation, are evaluated using a swallowing protocol three hours after extubation, and begin drinking if possible, Usual care group: fasting for six hours after extubation	Started two hours after extubation	Post-extubation to six hours post-extubation	↓	NA
Şavluk, 2017, Turkey [60]	RCT	Cardiac ICU	152 (Intervention group 1: 38/Intervention group 2: 37/Intervention group 3: 38/Control group: 39)	Patients who received a coronary artery bypass graft at scheduled surgery	Determine the impact of oral intake of carbohydrate-rich beverages before surgery in patients undergoing coronary artery bypass surgery	Thirst intensity: VAS; measured preoperatively, after preoperative induction, after postoperative ICU admission, six hours after ICU admission, and on postoperative day 1	Consumption of carbohydrate-rich beverages, Intervention group 1: 800 mL eight hours before surgery, 400 mL two hours before surgery; Intervention group 2: 400 mL eight hours before surgery; Intervention group 3: 400 mL two hours before surgery	Eight to 12 hours before surgery	Preoperative period	↓	NA
Lemiale, 2015, France [61]	RCT	4 ICUs	100 (HFNO group: 52/Venturi mask group: 48)	Age ≥18, patients with hypoxic acute respiratory failure and immunosuppression	Compare HFNO and Venturi mask oxygen in immunocompromised patients with acute respiratory failure	Thirst intensity: VAS; measured one and two hours after the start of oxygen therapy	HFNO group: oxygen administration with HFNO using a humidifier Venturi mask group: oxygen administration without humidification	Started at the beginning of oxygen therapy	Start of oxygen therapy two hours after	→	NA
Puntillo, 2014, United States [41]	RCT	SICU, cardiac ICU, neurological ICU	252 (intervention group: 127/Control group: 125)	RASS -1 to +1, ICU stay ≥24 hours, thirst intensity or thirst distress NRS ≥3	Test an intervention bundle for thirst intensity, thirst distress, and dry mouth in ICU patients	Thirst intensity: NRS, thirst distress: NRS, measured before and after intervention	Bundle for 15 minutes (3-4°C cold water swabs, cold water spray, mentholated moisturizer on lips)	Three times between 10:00 and 18:00	One to two days	↓	↓
Iblher, 2011, Germany [62]	RCT	Cardiac ICU	126 (Intervention Group 1: 25/Intervention Group 2: 25/Intervention Group 3: 24/Intervention Group 4: 27/Control group: 25)	After scheduled open chest surgery, on MV, sedation with propofol	Examine the influence of music intervention in the early postoperative period on patients undergoing open heart surgery	Thirst intensity: the ANP, measured on the third postoperative day	Intervention Group 1: 60 minutes of music with headphones immediately after ICU admission, Intervention Group 2: no music with headphones immediately after ICU admission, Intervention Group 3: 60 minutes of music on headphones immediately after sedation was discontinued, Intervention Group 4: no music with headphones immediately after discontinuation of sedation	Intervention group 1/2: start after ICU admission, Intervention group 3/4: start after sedation is discontinued	60 minutes	↑	NA
Feng, 2021, China [30]	Quasi-experimental study	SICU	183 (Observation 1 group: 61/Observation 2 group: 61/Control group: 61)	Postoperative gastrointestinal surgery, patients who were aware and able to communicate	Investigate the efficacy and feasibility of intermittent oxygen-driven humidification for the relief of thirst in postoperative gastrointestinal patients in the ICU	Thirst intensity: NRS; measured immediately after ICU admission, 10 minutes after admission, and four, eight, and 12 hours after admission	0.45% sodium chloride heated, 5 mL extracted, added to a spray inhaler mask, and humidified oxygen inhalation for 15 minutes (oxygen flow rate 6-8 L/min)	Group 1: immediately after admission and four hours later, Group 2: immediately after admission and four, eight, and 12 hours later	12 hours from ICU admission	↓	NA
Sharma, 2020, India [31]	Quasi-experimental study	Mixed ICU	40 (Experimental group: 20/Control group: 20)	Critically ill patients with thirst and dry mouth	Validate the effectiveness of the Thirst Bundle on thirst and dry mouth among patients admitted to the ICU	Thirst intensity: categorical Thirst Intensity Scale, measured before and after intervention	Intervention group: sterile cold water swab, sterile ice cold water, sterile menthol water spray	10 minutes for three consecutive days	Three days	↓	NA
Leemhuis, 2019, United States [42]	Prospective quality improvement project	SICU	136 (nurse intervention: 123/family intervention: 13)	―	Implement a research-based thirst intervention performed by ICU nurses and patients’ family members	Thirst intensity: NRS or Word Scale (0 to 3) or Yes/No, measured immediately before and after intervention	Bundle (repeated mouth swabs and sprays of ice-cold water and the application of a moisturizer containing spearmint, including menthol, to the lips and tongue)	Alternated the thirst intervention and the usual oral care	―	↓	NA
VonStein, 2019, United States [43]	Quasi-experimental study	Two MICUs	103 patient analysis (Intervention group: 62/Control group: 41)	ICU stay ≥ 12 hours, RASS -1 to +1, Thirst intensity or Thirst distress NRS ≥ 3, Assignment by unit	Evaluate the effectiveness of the scheduled use of ice water oral swabs and lip moisturizer with menthol compared with unscheduled use	Thirst intensity: NRS, thirst distress: NRS, measured seven hours after intervention	Intervention group (planned intervention): wiping with ice water oral swabs and applying mentholated moisturizing lip balm to lips, control group (unplanned intervention): unplanned implementation upon patient request	Every hour between 10:00 and 17:00	Seven hours	↓	↓
Noda, 2019, Japan [32]	Quasi-experimental study	SICU	38 (Intervention group: 28/Control group: 10)	Scheduled surgery, GCS: E4V5	Verify whether providing information about ICU environment to patients who are scheduled to be admitted to ICU in advance, using leaflets, will reduce their anxiety and environmental stress after admission	Original questionnaire about environmental stress, Stress level: 4-point Likert scale, measured in the general ward after discharge from ICU	Explain ICU (environment, lights out time, pain time, etc.) using leaflets with pictures and illustrations	Before surgery	Before surgery	NA	↓
Pagnucci, 2019, Italy [63]	Non-controlled pre-post study	SICU, EICU, and MICU	74	GCS ≥ 13, ICU stay ≥2 nights, Ramsay sedation score ≥2	Identify if complementary interventions affected conscious intensive care patients’ perception of stress factors and quality of sleep	Measured by SEDAICU, Degree of stress: 4-point Likert scale, Frequency of stressors: 3-point Likert scale, Measured on the first and second night	20 minutes of combined aromatherapy and massage care, and music or nature sounds throughout the night	Music or nature sounds: all night long, massage and aromatherapy: 20 minutes	All night long	NA	→
Oto, 2011, Japan [33]	Bench study	Mixed ICU	23 (NIV Heated Humidifier Setting Max group: 11/Med group: 12)	Age ≥20, patients with acute respiratory failure, NIV ≥24 hours	Investigate factors related to humidification during NIV	Thirst intensity: NRS, measured at the start of NIV, 12 and 24 hours later, and 12 and 24 hours after discontinuation of NIV	Max group: NIV heated humidifier set to maximum setting (Level 9 setting), Med group (Control group): Set to level 5	At the start of NIV use	NIV started - NIV stopped	→ (Med group:↑)	NA
Roca, 2010, Spain [64]	Prospective comparative study	Mixed ICU	20	Patients with acute respiratory failure, GCS:15	Compare the comfort of oxygen therapy via high-flow nasal cannula versus via conventional face mask in patients with acute respiratory failure	Thirst intensity: VAS, measured after each 30-minute intervention	Change to oxygen administration via high-flow nasal cannula after oxygen administration via face mask	SpO2 96% or higher with humidified oxygen administered by face mask	1 hour (0.5 hours each)	↓	NA
Nagashima, 2008, Japan [34]	Quasi-experimental study	Mixed ICU	22 (Method 1: 9/Method 2: 13)	Postoperative cardiovascular surgery, Patients with JCS single digits	Test the efficacy of salivary gland stimulation with a sponge brush as a method of relieving thirst in patients after cardiovascular surgery	Thirst intensity: 4-point Likert scale, measured after ICU discharge	Method 1: gargle with ice water, Method 2: stimulate salivary glands with care and moisturizing gel on lips	Method 1: when thirsty, Method 2: when regular and thirsty	Extubation: the start of drinking water	↓	NA

Prevalence of Thirst

**Figure 2:**
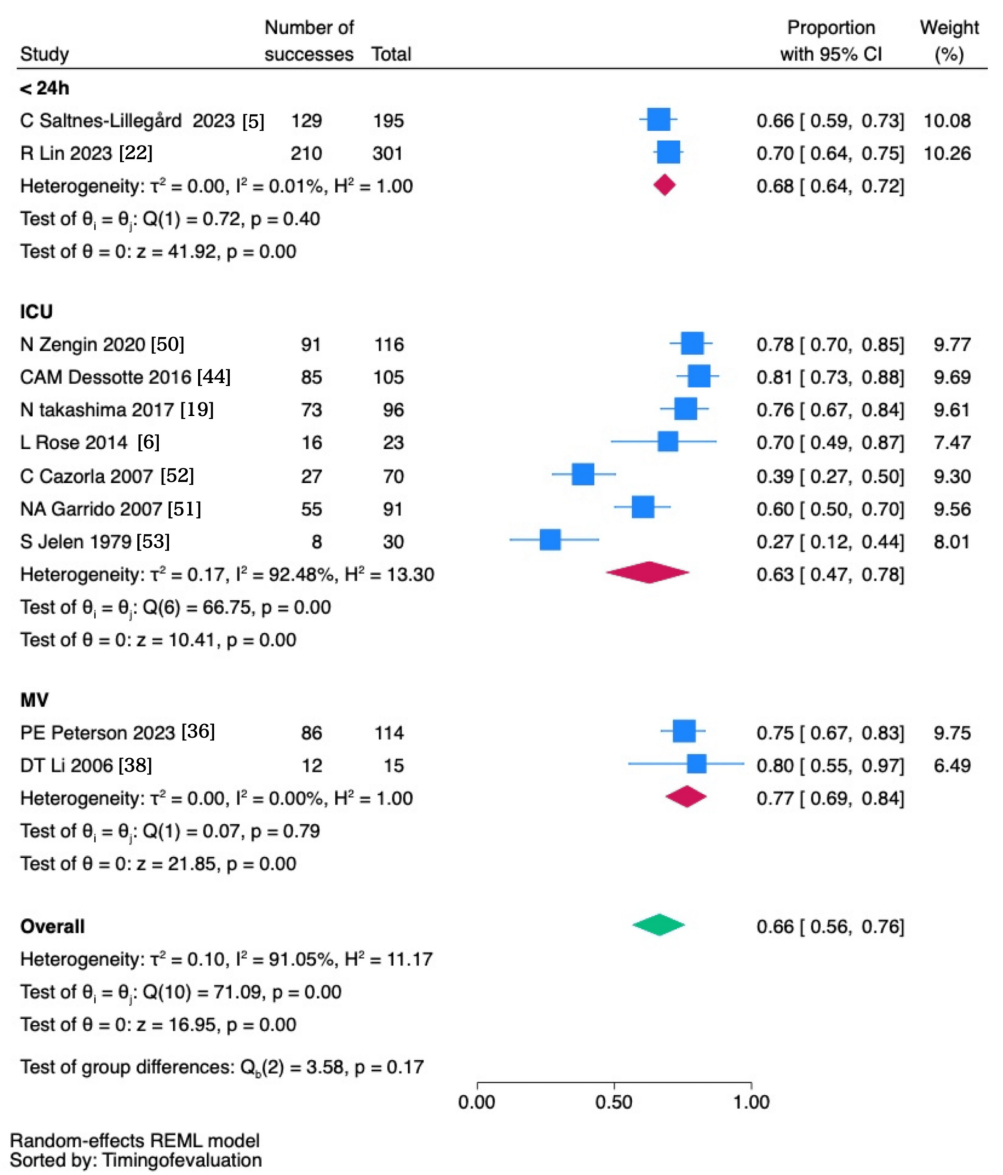
Prevalence of thirst symptoms in ICU patients

The prevalence of thirst was reported in 11 studies [5,6,19,22,36,38,44,50-53] and synthesized (Figure 2). Overall, the prevalence of thirst was 66% (95% CI: 56-76). The prevalence of thirst among ventilated patients was 77% (95% CI: 69-84). The prevalence of thirst within 24 hours of ICU admission was 68% (95% CI: 64-72), and during ICU stay was 63% (95% CI: 47-78).

Causes and Risk Factors for Thirst

Four studies [5,22,37,45] identified the risk factors for thirst using multivariable analysis. The reported risk factors were classified as patient/disease, treatment, and biochemical factors (Table 4).

**Table 4:** Risk factors for thirst reported in the included studies

Category	Risk factors for thirst (reference numbers)
Patient/disease factors	Sepsis diagnosis [5]
	Greater disease severity [22]
	Gastrointestinal diseases [37]
	Xerostomia [45]
Treatment factors	Analgesic administration [5]
	Surgery [22]
	Diuretics (such as high furosemide doses) [37,45]
	Selective serotonin reuptake inhibitors [37]
	Antihypertensive medications [37]
	High opioid doses [37]
	Negative fluid balance [37]
	Prohibition of oral intake [22,37,45]
	Mechanical ventilation [37]
	Use of a humidified Venturi mask [45] (compared with a nasal cannula)
Biochemical factors	High glucose [22]
	Low ionized calcium [37]
	Increasing serum sodium concentration [45]

Measurement Tools for Thirst and Its Intensity and Distress Level

The values measured in non-intervention studies are listed in Table 5. Thirty-six studies measured thirst intensity [4,5,8,10,11,18,21,22,24-31,33,34,36-43,45,54-56,58-62,64]. Using the Numerical Rating Scale (NRS), thirst intensity ranged from 5.37 to 8.34 (Table 5). Twenty-five studies [5,8,10,11,18,21,22,24-30,33,37,38,40-43,45,56,58,59] used the NRS to measure thirst intensity. Four studies [55,60,61,64] used the Visual Analog Scale (VAS). Other scales used included the Likert scale [4,34,35,39,62], word scale [42], and Thirst Intensity scale [31,54]. Two studies [22,42] used two or more scales to measure the thirst intensity. Twenty-four studies measured thirst distress and stress [4,5,10,19,20,23,25-27,32,35-37,41,43,44,46,48-51,56,57,63]. Using the NRS, thirst distress ranged from 3 to 6 (Table 5). Five studies [10,37,41,43,56] used the NRS, and three studies [23,48,57] used the VAS. Fourteen studies [4,5,19,20,27,32,35,36,44,46,49,50,51,63] used 3 to 14-point Likert scales. Other tools included the Thirst Distress Scale (TDS), which was used in two studies [25,26], and the TDS for patients with heart failure (TDS-HF), which was used in one study [56]. The TDS-HF consists of eight items rated on a 5-point Likert scale. One study [56] examined the correlation between NRS and TDS-HF using both scales.

**Table 5:** Measurement tools and values of thirst intensity and thirst distress and stress reported in the included studies

Category	First author (year)	Measurement tool	Value
Thirst intensity	Saltnes-Lillegård (2023) [5]	NRS, mean (95% CI)	6.13 (5.70-6.56)
	Flim (2022) [56]	NRS, median (IQR)	6 (3.0-8.0)
	Negro (2022) [45]	NRS, mean ± SD	5.37 ± 3.8
	Doi (2021) [8]	NRS, median (IQR)	6 (5-8)
	Krupa (2021) [54]	NRS, mean ± SD, median	8.34 ± 1.36, 8
	Puntillo (2010) [4]	3-point Likert scale, mean ± SE	2.16 ± 0.87
	Li (2006) [38]	NRS, mean ± SD	5.7 ± 3.7
Thirst distress and stress	Gunnels (2024) [35]	NRS, mean ± SD	3 ± 3.3
	Saltnes-Lillegård (2023) [5]	NRS, mean (95% CI)	4.98 (4.29-5.67)
	Film (2022) [56]	NRS, median (IQR)	6 (4.5-8.0)
	Karaer,(2021) [49]	4-point Likert scale, mean ± SD	2.79 ± 1.32
	Baumstarck (2019) [57]	VAS (0-100), mean ± SD	31 ± 35.04
	Takashima (2017) [19]	6-point Likert scale, mean ± SD	3.67 ± 1.4
	Dessotte (2016) [44]	5-point Likert scale, mean ± SD	2.6 ± 1
	Wang (2015) [23]	VAS (0-10), mean ± SE	5.789 ± 0.022
	Puntillo (2010) [4]	3-point Likert scale, mean ± SE	1.9 ± 0.80
	Kalfon (2010) [48]	VAS (0-100), mean ± SD, median	32 ± 34, 20
	Hweidi (2007) [20]	4-point Likert scale, mean ± SD	3.31 ± 0.79

Interventions for Thirst

**Figure 3:**
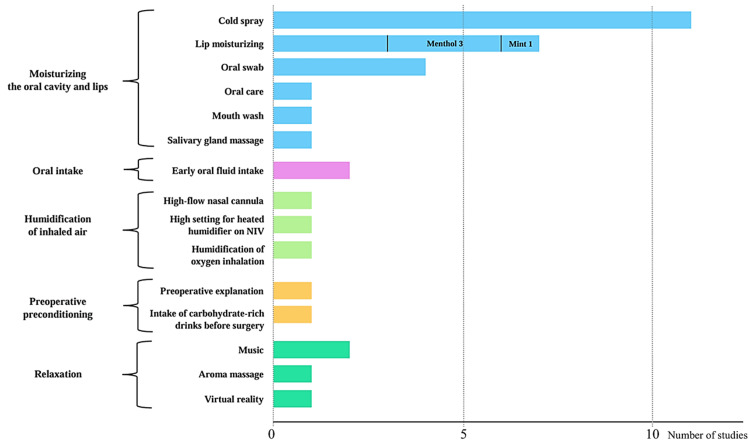
Graph of the type of intervention performed and the number of studies The total exceeds the number of studies, as each study included multiple interventions [10,11,24-34,40-43,58-64]. NIV, noninvasive mechanical ventilation

The effects of each intervention are shown in Table 3. The interventions and number of studies conducted for each method are illustrated in Figure 3. Seven studies implemented bundle [11,31,41,42] or package [10,26,43] interventions. These interventions mainly combined oral moisturization with sprays, swabs, and lip moisturization. Sprays included cold water [10,24,25,27,29,31,41,42,58], mint [26], and vitamin C [11]. Some studies compared the contents and temperatures of sprays [11,24,25,27]. Lip moisturizers include menthol [26,41,43], mint [42], and glycerin [11]. The timing of interventions varied; interventions were regularly scheduled in seven studies [10,11,25,31,41,42,43] and included both regular and as-needed interventions for thirst in others [27,29,34]. The intervention frequency varied from studies that intervened every 30 minutes over a three-hour period to those that intervened three times a day. Other interventions included early oral intake [28,40], humidification [30,33,61,64], preconditioning before surgery [32,60], and relaxation interventions [59,62,63]. Most interventions were effective in reducing thirst intensity and distress. However, music interventions [62], combined music and aroma interventions [63], and virtual reality [59] did not improve thirst. The high-flow nasal cannula oxygen therapy group showed no significant difference with respect to thirst compared with the Venturi mask group [61]. However, an observational study comparing face masks reported a reduction in thirst intensity [64].

Discussion

In this scoping review, we collected evidence on thirst in adult ICU patients. We included studies on the broad concepts of discomfort and stress among ICU patients, regardless of ICU stay duration or cause. Therefore, the evidence covers the overall state of thirst in ICU patients.

Prevalence and Impact of Thirst

The overall prevalence was 66%. It was high during mechanical ventilation, within 24 hours of ICU admission, and throughout the ICU stay. Studies conducted after 2010 have shown increased prevalence rates, possibly owing to increased recognition of thirst as a significant symptom and a shift to light sedation management. Thirst intensity was moderate to high in all studies and was reported to be the most distressing symptom among the common ICU symptoms (pain, thirst, anxiety, fatigue, and shortness of breath). Over 50% of respondents who experienced extreme ICU stress attributed this stress to thirst.

Measurement Tools and Methods for Thirst

Many studies have utilized the NRS, while others have used the VAS, Likert scale, and Thirst Intensity scale. These methods are effective for patients who can express their symptoms, but may overlook those who cannot. Most of the research has primarily focused on patients with Richmond Agitation and Sedation Scale scores ranging from -1 to +1 and those assessed as confusion-negative using the Confusion Assessment Method for the ICU. However, since many patients in the ICU are sedated or unconscious, there is a need to develop objective methods to assess the subjective thirst felt by patients. While oral dryness evaluation is one such method, it may miss patients’ perceived thirst because oral dryness does not always correlate with subjective thirst [8]. Additionally, conditions such as hypernatremia and elevated plasma osmolality have been associated with subjective experiences of thirst [2,65]. This indicates a need for further exploration of biochemical markers and the development of non-verbal measurement tools, which could significantly enhance the assessment of thirst in patients, particularly those who are unable to communicate their needs effectively. In addition, the assessment timing and frequency varied, with some studies evaluating registration, discharge, or daily care. Standardization of the timing, intervals, and frequency of assessments is required. Additionally, the criteria for when to intervene based on the NRS, VAS, or Likert scale scores have not yet been established. Recognizing the high prevalence of thirst among patients requires the development of standardized assessment protocols, measurement tools, and consistent timing.

Risk Factors for Thirst

The risk factors for thirst have been examined; however, direct intervention studies are few and unverified. These factors include patient/disease, treatment, and biochemical factors, which are related to ICU admission, diseases, and treatments [5,22,37,45]. Although patient/disease factors are unmodifiable, treatment and biochemical factors can be managed. Interventions such as avoiding hyperglycemia and correcting hypernatremia are relatively simple. Most interventions studied thus far are direct methods, such as ice water sprays. However, systemic management, addressing electrolyte imbalances and medication tapering, is also needed.

Intervention Methods and Effectiveness for Thirst

The effectiveness and sustained outcomes of interventions for managing thirst in ICU patients, including their optimal timing and frequency, remain largely ambiguous. This scoping review has identified that while various intervention methods exist, such as oral and lip moisturization through sprays and swabs with cold water, often implemented in bundles [11,31,41,42] or packages [10,26,43], the variability in methods, timing, and intervals complicates our understanding of their impact. ICU patients frequently cannot take oral fluids, restricting the amount of moisture applied. Some studies have indicated effectiveness with adjustments in water temperature [10,24,25,27,29,31,34,41,42,43,58] and the use of vitamin C water [11] or peppermint water [26]. Most studies included various interventions, but the effects and duration of a single intervention remain unclear. The duration of the intervention effects was investigated in only one study [8]. Thus, recognizing the absence of conclusive evidence for effective and lasting intervention methods highlights the critical need for further research. Additionally, there are numerous alternative interventions, such as preoperative measures [32,60], humidification adjustments of oxygen equipment [30,33,61,64], and relaxation techniques [59,62,63], each requiring further validation to confirm their efficacy.

Limitations

This study has several limitations. First, it focused on studies in which the primary concern was the patients’ perceived thirst, excluding studies that only examined dry mouth. Additionally, qualitative studies and gray literature were not included in our search, which may have affected the interpretation of prevalence rates and risk factors. Second, among the included studies, only four conducted multivariable analyses, which represents a significant limitation. This small number of studies with adjusted analyses limits our ability to draw definitive conclusions about independent risk factors and affects the generalizability of our findings. A larger study would increase the reliability of conclusions regarding risk factors. Furthermore, this scoping review did not assess the quality of the evidence. Therefore, the results and implications derived from the included studies should be interpreted with caution [16]. Despite these limitations, this scoping review provides valuable insights into the current state of thirst among ICU patients and identifies areas for future research development.

## Conclusions

Thirst is highly prevalent among ICU patients and is a significant cause of distress. However, standardized methods for evaluating thirst intensity and distress remain unclear, particularly for patients who are unable to self-report, because no objective measurement tools have been established. Additionally, there are no defined thresholds for initiating thirst intervention. Although various interventions such as cold or mint water sprays or swabs have been explored, evidence on the most effective and sustained approaches, including their timing, frequency, and removal of risk factors, remains insufficient. Future studies should focus on validating assessment methods, including the development of objective measurement tools, and optimal intervention methods, including the removal of the risk factors for thirst identified in this study.

## Appendices

Appendix A

**Table 6:** Search terms

Database	Search terms
PubMed	#1 thirst [mh] OR xerostomia[mh] OR thirst*[tiab] OR xerostomia[tiab] OR "dry mouth"[tiab]OR "sensation of dryness"[tiab] OR "mouth dryness"[tiab] OR ice[mh] OR menthol[mh] OR "cold oral stimul*"[tiab] OR ice[tiab] OR "cold water"[tiab] OR gargl*[tiab] OR "wet gauze"[tiab] OR wipe*[tiab] OR menthol*[tiab] OR "lip moisturizer"[tiab] OR "citric acid spray*"[tiab] OR "oral care"[tiab] #2 Intensive care units[mh] OR critical care[mh] OR Critical illness[mh] OR "ICU"[tiab] OR"intensive care unit*"[tiab] OR "intensive care"[tiab] OR "critical care"[tiab] OR"critical illness"[tiab] OR "critically ill"[tiab] #3 animals [mh] NOT humans [mh] #1 AND #2 NOT #3
CINAHL	#1 MH thirst OR MH xerostomia OR thirst* OR xerostomia OR "sensation of dryness" OR "mouth dryness" OR "dry mouth" OR MH ice OR "cold oral stimul*" OR "cold water" OR gargl* OR "wet gauze" OR wipe OR menthol* OR "lip moisturizer" OR "citric acid spray" OR "oral care" #2 MH Intensive care units OR MH critical care OR MH Critical illness OR "ICU" OR "intensive care unit" OR "intensive care" OR "critical care" OR "critical illness" OR "critically ill" #3 animals [mh] NOT humans [mh] #1 AND #2 NOT #3
CENTRAL	#1 MeSH descriptor:[critical care]explode all trees OR MeSH descriptor:[intensive care unit]explode all trees OR MeSH descriptor:[critical illness]explode all trees OR "critical care":ti,ab,kw OR "intensive care unit":ti,ab,kw OR ICU:ti,ab,kw OR "critical illness":ti,ab,kw OR "critically ill":ti,ab,kw #2 MeSH descriptor:[thirst]explode all trees OR MeSH descriptor:[xerostomia]explode all trees OR thirst*:ti,ab,kw OR xerostomia:ti,ab,kw OR "dry mouth":ti,ab,kw OR "sensation of dryness":ti,ab,kw OR "mouth dryness":ti,ab,kw OR MeSH descriptor:[ice]explode all trees OR MeSH descriptor:[menthol]explode all trees OR ice:ti,ab,kw OR menthol*:ti,ab,kw OR "cold oral stimul*":ti,ab,kw OR "cold water":ti,ab,kw OR gargl*:ti,ab,kw OR "wet gauze":ti,ab,kw OR wipe:ti,ab,kw OR "lip moisturizer":ti,ab,kw OR "citric acid spray":ti,ab,kw OR "oral care":ti,ab,kw #3 animals:ti,ab,kw NOT humans:ti,ab,kw #1 AND #2 NOT #3
The Igaku Chuo Zasshi	#1 渇き/TH OR 口内乾燥症/TH OR 口渇/TA OR 渇き/TA OR 口腔乾燥症/TA OR ドライマウス/TA OR氷/TH OR 含嗽/TH OR 冷水/TH OR Menthol/TH OR 氷/TA OR 含嗽/TA OR 冷水/TA OR Menthol/TA OR うがい/TA OR 口腔ケア/TA OR リップクリーム/TA OR Lip Cream/TA OR メントール/TA OR メンソール/TA OR 洗口/TA OR ((Citric Acid/TA OR クエン酸/TA) AND (エアゾール/TA OR スプレー/TA)) #2 ICU/TH OR クリティカルケア/TH OR 危篤/TH OR ICU/TA OR 集中治療室/TA OR 集中治療/TA OR クリティカルケア/TA OR 危篤/TA OR 重症/TA OR 重病/TA OR重症疾患/TA #3 動物/TH NOT ヒト/TH #1 AND #2 NOT #3
